# Flavonoids as Therapeutic Agents in Alzheimer's and Parkinson's Diseases: A Systematic Review of Preclinical Evidences

**DOI:** 10.1155/2018/7043213

**Published:** 2018-05-10

**Authors:** Roxana Braga de Andrade Teles, Tâmara Coimbra Diniz, Tiago Coimbra Costa Pinto, Raimundo Gonçalves de Oliveira Júnior, Mariana Gama e Silva, Érica Martins de Lavor, Antonio Wilton Cavalcante Fernandes, Ana Paula de Oliveira, Fernanda Pires Rodrigues de Almeida Ribeiro, Amanda Alves Marcelino da Silva, Taisy Cinthia Ferro Cavalcante, Lucindo José Quintans Júnior, Jackson Roberto Guedes da Silva Almeida

**Affiliations:** ^1^Postgraduate Program in Biotechnology, State University of Feira de Santana, 44036-900 Feira de Santana, BA, Brazil; ^2^Federal University of San Francisco Valley, 56304-205 Petrolina, PE, Brazil; ^3^Postgraduate Program in Neuropsychiatry and Behavioural Sciences, Federal University of Pernambuco, 50740-521 Recife, PE, Brazil; ^4^UMRi CNRS 7266 LIENSs University of La Rochelle, La Rochelle, France; ^5^University of Pernambuco, 56328-903 Petrolina, PE, Brazil; ^6^Department of Physiology, Federal University of Sergipe, 49100-000 São Cristóvão, SE, Brazil

## Abstract

Alzheimer's and Parkinson's diseases are considered the most common neurodegenerative disorders, representing a major focus of neuroscience research to understanding the cellular alterations and pathophysiological mechanisms involved. Several natural products, including flavonoids, are considered able to cross the blood-brain barrier and are known for their central nervous system-related activity. Therefore, studies are being conducted with these chemical constituents to analyze their activities in slowing down the progression of neurodegenerative diseases. The present systematic review summarizes the pharmacological effects of flavonoids in animal models for Alzheimer's and Parkinson's diseases. A PRISMA model for systematic review was utilized for this search. The research was conducted in the following databases: PubMed, Web of Science, BIREME, and Science Direct. Based on the inclusion criteria, 31 articles were selected and discussed in this review. The studies listed revealed that the main targets of action for Alzheimer's disease therapy were reduction of reactive oxygen species and amyloid beta-protein production, while for Parkinson's disease reduction of the cellular oxidative potential and the activation of mechanisms of neuronal death. Results showed that a variety of flavonoids is being studied and can be promising for the development of new drugs to treat neurodegenerative diseases. Moreover, it was possible to verify that there is a lack of translational research and clinical evidence of these promising compounds.

## 1. Introduction

For many years, neurodegenerative diseases such as Alzheimer's and Parkinson's diseases have been a major focus of neuroscience research to understanding the cellular alterations and pathophysiological mechanisms [1]. Neurodegenerative diseases are multifactorial conditions in which many biological processes become unregularly [2] mediated by endogenous, genetic, and environmental factors [3] intimately associated with progressive brain damage [4].

The generation of free radicals and oxidative stress producing cellular impairments is often cited as an important factor in the etiology of neurodegenerative diseases [5, 6]. Beyond the oxidative stress, the neurodegenerative disease pathogenesis has some common characteristics such as neuroinflammation, abnormal accumulation of proteins, and aging [7–9].

Alzheimer's disease (AD) is a chronic progression characterized by loss of memory and cognitive deficits such as agnosia, aphasia, and apraxia, among others, facts that cause interference in daily life and in the individual's work. The prevalence rate is about 7% for individuals aged 65 or more, with the risk doubling every 5 years [10].

Parkinson's disease (PD) is the second most common neurodegenerative disease after Alzheimer's. PD is a chronic neurodegenerative disease characterized by the loss of dopaminergic neurons in the substantia nigra which leads to decreased levels of dopamine in the striatum and disrupted motor control [11, 12]. Its incidence is usually comprised between 10 and 50/100,000 person-years, and its prevalence between 100 and 300/100,000 population and prevalence both increase progressively after 60 years of age [13].

Recently, studies showed considerable efforts on search of antioxidant plant-derived polyphenol compounds with neuroprotective potential for the treatment of neurodegenerative diseases [14]. Dietary flavonoids have been suggested to prevent and treat neurodegenerative diseases [9].

Flavonoids are found in numerous plants, fruits, and vegetables [15] and are known as the most common phytochemicals which possess a multiple range of pharmacological effects [16]. These secondary metabolites have been described as potent antioxidant, free radical scavengers, and metal chelators [4–17], also presenting anticholinesterase [18], antiaging [7], neuroprotective [2, 19] and anti-inflammatory properties [5, 20, 21], and neurotrophic roles [22], ameliorating learning and memory [23], possessing potent antidepressant and antiamyloidogenic effects [16], suppressing the activation of microglia, and mediating inflammatory processes in the central nervous system (CNS) [24]. Moreover, flavonoids are able to cross the blood-brain barrier with chronic or acute administration suggesting that these compounds can feasibly have a direct effect on the brain, so this chemical compounds could be used as a prophylactic, in order to slow down the progression of diseases such as AD and PD [11].

Considering that neurodegenerative diseases represent some of the greatest challenges for basic science and clinical medicine, our study consists a systematic review of pharmacological reports involving the use of flavonoids on neurodegenerative conditions, especially AD and PD.

## 2. Materials and Methods

### 2.1. Search Strategy

The present systematic review was conducted according to the guidelines for Transparent Reporting of Systematic Reviews and Meta-Analyses (PRISMA statement) [25]. In this review, the specialized databases Pubmed, Web of Science, “Biblioteca Regional de Medicina” (BIREME), and Science Direct were used for the literature search in September and October 2017, using the terms “flavonoids, bioflavonoids, flavonols, flavonoid, anthocyanin, flavone, flavones, 2-phenyl-benzopyrans, 2-phenyl-chromenes, isoflavonoids or flavonones,” combined with “neurodegenerative diseases, Alzheimer disease or Parkinson disease.” We did not contact investigators, nor did we attempt to identify unpublished data.

### 2.2. Study Selection

In this step, two independent researchers (T.C.C.P. and T.C.D.) conducted the selection, and electronic search titles, selected abstracts, and full-text articles were independently reviewed by a third researcher who conducted the analysis of the full text. Disagreements on study inclusion/exclusion were resolved with a consensus between all investigators. The following inclusion criteria were applied: studies with flavonoids acting on CNS in animal models. Studies were excluded according to the following exclusion criteria: review articles, meta-analyses, abstracts, conference proceedings, editorials/letters, case reports, studies in humans, and published over 5 years. Additional papers were included in our study after analyses of all references from the selected articles.

### 2.3. Data Extraction

Data were extracted by one reviewer using standardized forms and were checked by a second reviewer. Extracted information included the substance, animal models, doses and concentrations, and evaluated parameters.

### 2.4. Methodological Quality Assessment

The risk of bias and quality of preclinical evidences were assessed using a checklist adapted from Hooijmans et al. [26] and Siqueira-Lima et al. [27]. This analysis allowed evaluating the methodological quality of the selected manuscripts regarding the outcome measurements, randomization of treatment allocation, blinded drug administration, blinded outcome assessment, appropriate description of the doses and routes of administration used, and appropriately reported statistical analysis of data. Studies that reported randomization of animals, blinding, outcome measurements, and statistical analysis were considered to be of higher methodological quality.

## 3. Results and Discussion

### 3.1. Study Selection

**Figure 1:**
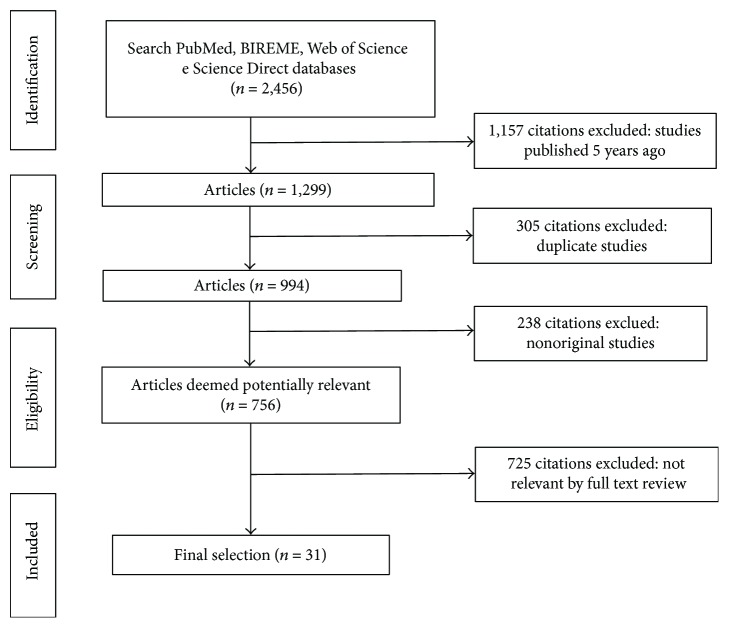
Flowchart for the literature searching and screening.

The primary search identified 2456 articles, including 347 from PubMed, 1338 from BIREME, 595 from Web of Science, and 176 from Science Direct. Among these, 1157 documents were published more than five years ago and were therefore excluded from the review. In addition, 305 manuscripts were indexed in two or more databases and were considered only once, resulting in 994 eligible articles. After an initial screening of titles and abstracts, followed by a full text analysis, 31 articles were included in this systematic review, while the reminder (*n* = 728) was excluded since they did not meet the inclusion criteria. A flowchart illustrating the progressive study selection and numbers at each stage is shown in Figure 1.

### 3.2. Characteristics of Included Studies

In general, the studies were conducted by research groups from several countries, mainly China (10 reports, 32.3%), Republic of Korea (07 reports, 22.6%), and India (05 reports, 16.1%). These findings reflect the contribution of Oriental medicine in the search for new drugs from natural products. In fact, Ayurveda and Traditional Chinese Medicine (TCM) are major traditional treatment systems used not only in India and China, but also in several countries [28, 29]. These systems have provided important information for the development of new pharmaceutical products based on plant extracts or even small molecules of original chemical structure and with innovative mechanisms of action. Their by-products have been applied in a variety of diseases, including central nervous system disorders such as AD and PD [30, 31].

Chinese herbal medicines have been clinically used to treat AD for a long time with significant effectiveness [32]. Recently, Jiang et al. [33] have described the progress of TCM in the treatment of AD, highlighting traditional Chinese medicinal herbs as potential AChE inhibitors for anti-AD approach. Among bioactive molecules, numerous flavonoids have been cited, including quercetin, apigenin, epigallocatechin-3-gallate, catechin, epigallocatechin, epicatechin-3-*O*-gallate, icariin, procyanidin, and silibinin. Similarly, TCM has also been reported for the treatment of PD. Several Chinese plant derivatives have been shown to be promising neuroprotective agents in PD, including resveratrol, curcumin, and ginsenoside [34, 35]. In this context, such information justifies the significant number of Chinese authorship studies included in this review.

Although most of the manuscripts was focused on AD (20 reports), a relevant number of studies involving experimental protocols in PD were also found (13 reports). Many studies have evaluated important behavioral parameters related to memory, motor functions, and cognitive activities of animals. However, biochemical and molecular targets were also verified by colorimetric and enzymatic assays; histological, biochemical, and hematological analyses; and western blot, immunohistochemical, and immunofluorescence techniques. The parameters evaluated and main outcomes are summarized in Table 1.

### 3.3. Methodological Quality

**Figure 2:**
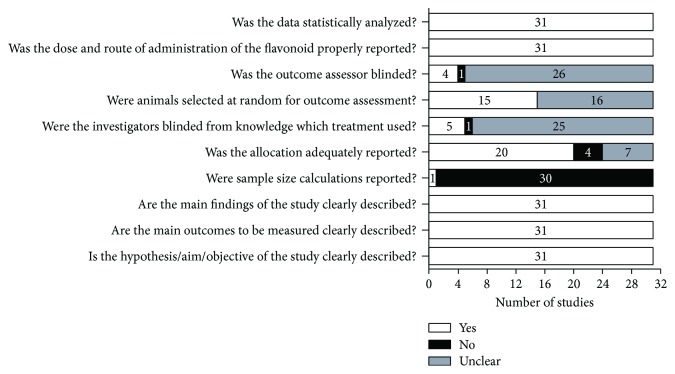
Methodological quality of included studies. *Light bars* indicate the proportion of articles that met each criterion; *dark bars* indicate the proportion of studies that did not; and *white gray bars* indicate the proportion of studies with unclear answers.

Concerning the methodological quality, all reports were carefully evaluated through a standard checklist. As shown in Figure 2, only one of the included articles reported sample size calculations. Only 48.4% of the studies reported randomization of animals for outcome assessment. Allocation of the animals in different treated groups was adequately reported by 64.5% of the studies. Blinding of investigators during animal treatment and outcome evaluation was found only in 16.1 and 12.9% of the studies, respectively. The remaining studies did not report any blindness strategy.

Although these parameters are most often applied in clinical trials, the need of randomization, blinding, allocation concealment, and attempt to minimize variation has been discussed and strongly recommended for preclinical studies, especially when the experimental protocols involve the evaluation of behavioral parameters. Since human studies are often justified based on results from animal studies, it is extremely important to plan preclinical protocols that follow the same rigor of clinical trials. In addition to reducing the risk of bias, this research strategy avoids obtaining distinct results between the two study designs (preclinical and clinical) [67–69]. In this context, the methodological quality of the studies included in our review was considered low to moderate, which limits the interpretation of the results.

### 3.4. Animal Models in Alzheimer's Disease

AD is a gradual and highly prevalent neurodegenerative disease and has as pathological neuroinflammation characteristic, neuronal loss, and impairment of cognitive function [47, 70]. AD is classified into two subtypes: early-onset familial outcome, related to specific mutations in genes that code for presenilin 1 (PS1), presenilin 2 (PS2), and amyloid precursor protein (APP), and late-onset sporadic disease, associated with mutations in genes that code for apolipoprotein E (ApoE), which include several environmental and genetic risk factors, yet unknown [71].

Among the pathological features, there is an amyloid dense core of *β*-amyloid peptide (A*β*) and intracellular neurofibrillary tangles (NFTs) composed of an abnormally phosphorylated form of the tau protein [41]. The deposition of amyloid *β* peptide culminates in pathological processes of synaptic and cognitive dysfunction and neuronal death [47, 70].

Animal models recapitulate pathological feature characteristic of AD in addition to several phenotypic traits simulate of the disease, acting as therapeutic targets and in their preclinical validation. According to Laurijssens et al. [72], animal models used in AD can be divided into three categories: natural (dog, mouse lemur, Octodon, and Rhesus monkey), genetic (APP mice, Tg2576 mice, and PSAPP mice), and interventional models (rat, intrahippocampal amyloid infusion model).

In the selected studies, interventional models were the most used [39, 44, 47, 49, 51, 53]. In fact, interventional models would generally be better at identifying symptomatic or corrective treatment. These models can provide important insights such as the A*β* pharmacochemical substance-induced model and the understanding of inflammation, neurotoxicity, neurodegeneration and synaptic function, specific brain lesions, and neuronal mechanism underlying memory dysfunction [72, 73].

### 3.5. Animal Models in Parkinson's Disease

PD is a progressive neurodegenerative disease defined by the selective loss of dopaminergic neurons in the nigrostriatal pathway [62]. A disruption of synaptic activity may represent the primary event in PD pathogenesis. Recently, studies on the neurodegeneration mechanisms in PD have revealed pathological features and important genetic influences. Common pathogenic pathways are present in PD, such as proteostatic deficits, mitochondrial dysfunctions, oxidative stress, and inflammation process [74, 75].

As well as knowledge of new pathophysiological mechanisms involved, the development of experimental models in PD has also advanced. As in other neurodegenerative disorders, PD experimental protocols have become increasingly sensitive and accurate, allowing not only the evaluation of new drugs, but also the understanding of the molecular mechanisms that play an important role in the disease [74]. However, it is important to note that animal models still cannot express the complexity of human pathological hallmarks and clinical features, each one has specific limitations, and its choice must be conditioned to what best responds to the research objective [74, 76].

The papers selected in this review reported animal models to evaluate pathogenic mechanisms, motor and nonmotor manifestations of PD by administering different neurotoxic agents such as 6-hydroxydopamine (6-OHDA) [57, 58, 61, 65], 1-methyl-4-phenyl-1,2,3,6-tetrahydropyridine (MPTP) [56, 59, 62], or rotenone [63, 64]. The most cited animal model in the selected studies was 6-OHDA. It exerts toxic effects on catecholaminergic neurons and is characterized as a neurotoxin structural analogue of catecholamines, dopamine, and noradrenaline. It is toxic at the peripheral and central levels although its toxicity in the CNS is only possible through direct stereotaxic surgery. Its neurotoxicity involves the accumulation of toxin in the catecholaminergic neurons triggering the alteration of the cellular homeostasis and neuronal damages. 6-OHDA oxidation by MAO-A generates H_2_O_2_ which, besides being highly cytotoxic, triggers the production of oxygen radicals [77–79]. Another model included the alteration of dopaminergic neurotransmission by drugs, such as reserpine [80], or by genetic manipulation to study the progression of dopaminergic cell degeneration and motor signs [81].

The models that use toxins are widely used and act in the replication of most pathological and phenotypic features of the disease. However, as a limitation of this tool, the loss of approximately 70 to 80% of the dopaminergic neurons in the acutely induced neurodegeneration is difficult to achieve, making it difficult to explore dysfunction and progression. In view of the early synaptic dysfunction and neurodegeneration that are inherent to the pathology, new models are necessary to help the early stages of the disease, as well as new therapeutic strategies for the treatment of PD [76].

### 3.6. Flavonoids in the AD and PD Treatment

The plants use adaptation systems in extreme conditions, such as high temperatures, lighting, and water scarcity, which culminate in oxidative damages leading to excessive production of free radicals. Among the main natural antioxidant agents, we highlight carotenoids, flavonoids, anthocyanins, and phenolic derivatives that act in several mechanisms against oxidative stress [82].

Flavonoids are a class of secondary metabolites that always attract the attention of the pharmaceutical industry due to their versatility in therapeutic properties. Additionally, they are part of the human diet due to their abundance in vegetables, fruits, seeds, and beverages such as coffee, tea, and red wine, as well as in medicinal herbs. More than 9000 different flavonoids have been identified and are divided into six subclasses based on their molecular structure. These subclasses include flavonols (rutin, quercetin), flavanols (catechin, epicatechin, and epigallocatechin), isoflavones (genistein, daidzein, glycetin, and formanantine), anthocyanidins (cyanidin, malvidine, and delphinidine), flavanones (hesperetin, naringenin), and flavones (apigenin, luteolin) [83].

Studies indicate that phenolic compounds play an important role in prevention and treatment of age-associated neurodegenerative diseases. Among the neuroprotective actions of dietary flavonoids, we highlighted their potential to modulate cell signaling pathways, protect neurons against oxidative stress, inhibit nicotinamide adenine dinucleotide phosphate (NADPH) oxidase, subsequently decrease reactive oxygen species production, and downregulate proinflammatory transcription factors, as well as its ability to suppress neuroinflammation through reduction of the release of cytokines [84].

**Figure 3:**
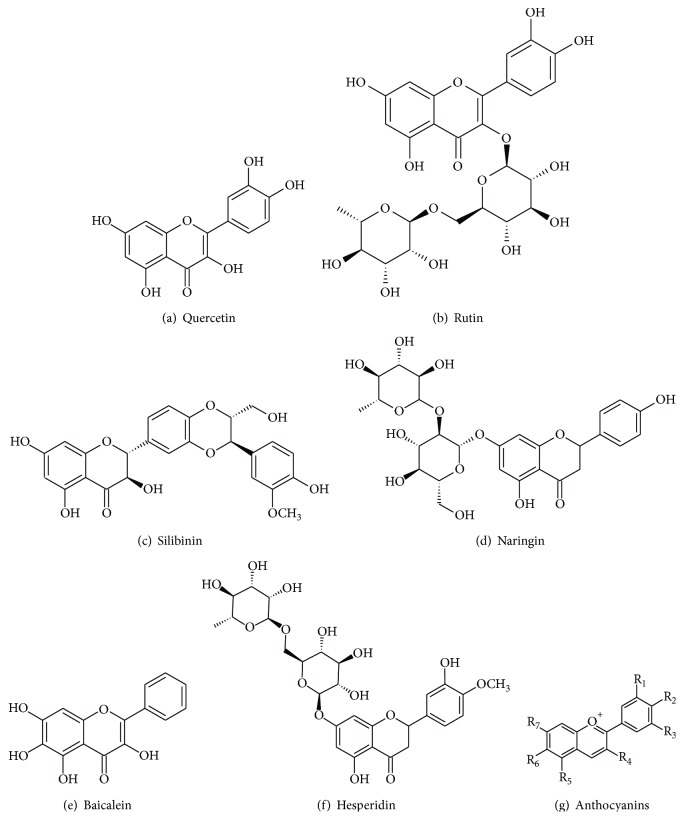
Chemical structures of flavonoids most cited in this review.

**Figure 4:**
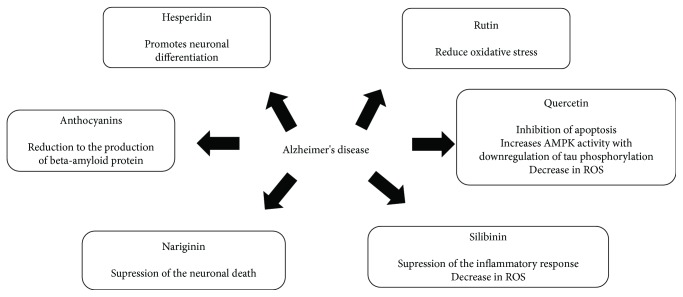
Possible mechanisms of action of flavonoids against Alzheimer's disease.

**Figure 5:**
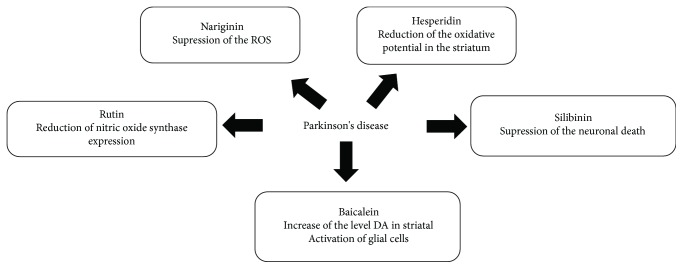
Possible mechanisms of action of flavonoids against Parkinson's disease.

In our systematic review, quercetin, rutin, silibinin, naringin, baicalein, hesperidin, and anthocyanins (Figure 3) were the most studied flavonoids for PD and AD treatment. In this sense, we describe below the pharmacological effects of these compounds to better understand the role of flavonoids in the treatment of AD and PD (Figures 4 and 5, resp.). In general, they act to improve the progression of the disease, but in the included reports, there is no consensus of dose, treatment duration, and route of administration of these flavonoids. However, the researchers argue that they have good bioavailability.

#### 3.6.1. Quercetin

Quercetin (3,3′,4′,5,7-pentahydroxyflavone) is a flavonoid found naturally in plants and among other foods such as onions, apples, broccoli, and red wine. It presents pharmacological, antioxidant, cardioprotective, and antiapoptotic properties [85]. Some studies relate their neuroprotective action in the prevention of neurodegenerative diseases like AD and PD [86]. Sharma et al. [54] evaluated the effect of quercetin manipulation in young rats treated with aluminum on isolated mitochondrial hippocampus. A decrease in the levels of reactive oxygen species (ROS) was observed; however, there were no significant changes in mitochondrial superoxide dismutase activity (MnSOD) [54].

Expression of the apoptotic markers in the mitochondrial fraction revealed levels of reduced Bax and increased Bcl. When the cytosolic fractions were evaluated, the expression of Bcl-2 and Cyt c was decreased, reducing the activation of caspase-3 suggesting that the administration of quercetin inhibits apoptosis. Quercetin prevents the release of Cyt c and subsequent activation of caspase-3 and decreases p53 expression. The histopathological analysis revealed no significant degeneration [54]. Thus, the study performed by Sharma et al. [54] has shown that quercetin attenuates aluminum-induced mitochondrial turgor, loss of ridge, and chromatin condensation.

Environmental factors such as hyperlipidemic diet consumption may increase the inflammatory response in the brain by increasing the release of cytokines such as TNF-*α* and IL-6 [87]. Studies have shown that the action of these cytokines promotes neuronal death by activating the apoptotic cascade [88]. Quercetin is also involved in the activity of AMPK, an energy sensor, which has the ability to prevent the phosphorylation of tau protein [89]. Tau protein facilitates the polymerization of tubulin in the cell, resulting in microtubule formation. In the neurofibrillary tangles, the aggregation of Tau is by irreversible phosphorylation suffered by this protein. This prevents its normal function and at the same time facilitates its aggregation in fibrils, preventing the normal functioning of the neuron [90].

Chen et al. [46] evaluated whether the activation of AMPK through the action of quercetin is able to block or not the phosphorylation of tau protein in animals submitted to a hyperlipidic diet. It was seen that quercetin was able to promote downregulation in blood levels of TNF-*α* and IL-6. And it especially prevented tau phosphorylation by restoring AMPK activation. Additionally, quercetin inhibits GSK3*β* enzyme activity by dephosphorylation, attenuates the expression of P-PERK and P-IRE1*α* (membrane sensors present in the endoplasmic reticulum whose activation indicates oxidative stress), and normalizes the inflammation of NLRP3 (inflammation promoter). Improvement in learning and spatial memory was also seen [46].

In a study that evaluated the manipulation of quercetin nanoparticle facilitating oral absorption, it was seen that there was no change in coordination or locomotor activity [48]. The nanoparticle treatment of quercetin is capable of promoting reversal of abnormal exploratory behavior by improving learning and memory assessed by the Morris water maze test. In addition, the expression of an inflammatory marker GFAP (glial fibrillary acidic protein) in the hippocampus was significantly reduced, which was not observed with free quercetin. On the other hand, the expression of CD11b, a member of the complement cascade, whose function is adhesion and leukocyte migration in response to inflammation, did not present significant results. Therefore, manipulation of quercetin-loaded nanoparticles has been shown to increase the concentration of this flavonoid in the brain of animals [48].

In the study performed by Palle and Neerati [50], the protective effect of quercetin nanoparticles compared to free quercetin against the induction of spatial memory deficiency promoted by scopolamine in animals was evaluated. This work highlighted a relevant role of quercetin nanoparticles on oxidative stress, revealing that these nanoparticles are able to significantly reduce malonaldehyde levels and increase levels of glutathione peroxidase and catalase in the brain. In observing the effect of treatment of quercetin nanoparticles on the pharmacological action of scopolamine, it has been seen that they are able to reduce the induction of elevation of scopolamine-promoted anticholinesterase activity. In addition, treatment with quercetin nanoparticles significantly reduces morphological abnormalities revealing the cellular protective effect of this flavonoid [50].

It is believed that changes in mitochondrial activity are the main cause of the occurrence of neurodegenerative disorders because they are the main producers of ROS [76]. Godoy et al. [49] have observed that quercetin exhibits antioxidant activity protecting against neuronal toxicity induced by hydrogen peroxide, although this protection has been partial in rat hippocampal neurons. Animals treated with quercetin caused a reduction in ROS levels, recovered normal mitochondrial morphology, and prevented mitochondrial dysfunction in neurons that were manipulated with hydrogen peroxide [49].

Of all the works selected in this review, only one demonstrated the role of quercetin on Parkinson's disease. Mu et al. [61] investigated the antitremor effect of quercetin in the experimental model of PD induced by the application of 6-hydroxydopamine (6-OHDA). The study demonstrated that injection of 6-OHDA into the striatum induces marked decrease in serotonin levels and its metabolite 5-hydroxyindole-3-acetic acid (5-HIAA). Therefore, it was seen that these animals when treated with quercetin had attenuated serotonin levels, which may be related to an improvement in the tremor level of these animals [61].

Quercetin seems to act by inhibiting the cell oxidative potential, in addition to exhibiting anti-inflammatory action. Both events may minimize the progression of PD and AD.

#### 3.6.2. Silibinin

Silibinin, (2*R*,3*R*)-3,5,7-trihydroxy-2-[(2*R*,3*R*)-3-(4-hydroxy-3-methoxyphenyl)-2- (hydroxymethyl)-2,3-dihydro-1,4-benzodioxin-6-yl]-2,3-dihydro-4*H * -chromen-4-one, is a flavonoid derived from milk thistle of the species *Silybum marianum* [91]. Recent research investigated the effects of silibinin on the inflammatory process, oxidative stress, and autophagy [92, 93]. Following this subject, Song et al. [47] investigated the effect of silibinin treatment on locomotor activity, learning, and spatial memory. In addition to the concentration of the proinflammatory cytokines IL-1*β*, IL-4, and the levels of the antioxidant enzyme (GSH) and malondialdehyde (MDA), a lipid peroxidation marker, which occurs in response to excess free radicals the NF-*κ*B (nuclear factor kappa B), a regulator of the immune response released in different situations among these oxidative stress, cyclooxygenase-2 (COX-2), i-NOS (nitric oxide synthase) are products of glial cells contributing to an inflammatory response in the brain. P-53, a tumor suppressor agent, is a critical component of the acute stress cell response, and p-p53 is its phosphorylated component. It has been seen that silibinin decreases anxiety-like behavior, reverses memory damage and spatial learning caused by the treatment of A*β*25-35, and improves the ability to recognize new objects and memory flexibility. Silibinin is able to suppress the inflammatory response and improve oxidative stress levels in the hippocampus, in addition to suppressing the expression of p-p53 and p-53 [47].

Chen et al. [60] analyzed the levels of glutathione and malondialdehyde in animals that underwent neonatal manipulation with carbonyl iron dose and its consequence in young adult and old animals as well as treatment with silibinin. It was first seen that iron consumption resulted in abnormal behavior of coordination and locomotor activity only in the old animals. Also in these animals, iron in the neonatal period also increased levels of malondialdehyde and reduced those of glutathione enzyme. Treatment with silibinin in aging animal's decrease dopamine depletion in the striatum improving motor behavior was also able to significantly reduce the content of MDA and increase the content of GSH in the nervous system. It is concluded that silibinin acts as a neuroprotective factor preventing oxidative stress; one of the consequences of neurodegenerative diseases, among them, is Parkinson's disease [60].

Using a model of dopaminergic neuronal death caused by the pharmacological agent MPTP (1-methyl-4-phenyl-1,2,3,6-tetrahydropyridine), a neurotoxin capable of inducing Parkinsonism, Lee et al. [62] investigated the neuroprotective mechanism of sibilinin. Treatment with silibinin prevented motor dysfunction in the pharmacological parkinsonism (MPTP) model, as well as neuronal loss (TH-positive neurons) in the striatum and substantia nigra. Silibinin effectively protects dopaminergic neurons in the striatum and black matter of neuronal death caused by MPTP, but does not impede the inflammatory response, since it does not act effectively on glial cell activation or modulation of oxidative stress. The latter result differs from the previous work probably due to the dose difference used in the present study, from 1 to 10 mg/kg body weight. This demonstrates that the neuroprotective action of silibinin is dose dependent [62].

It can be concluded that silibinin is a flavonoid with a neuroprotective characteristic especially acting on the inflammatory response and on the components of oxidative stress and neuronal death, thus having a potential therapeutic agent in neurodegenerative diseases.

In summary, silibinin is a flavonoid with a neuroprotective potential, especially acting on the inflammatory response, on components of oxidative stress, and on neuronal death.

#### 3.6.3. Anthocyanins

Anthocyanins are polyphenolic flavonoids widely found in fruits, flowers, grains, and vegetables [94]. This flavonoid has antioxidant, anti-inflammatory, and antiapoptosis properties, in addition to improving memory and cognition [95]. They are characterized by the basic flavylium core (2-phenylbenzopyryl cation) consisting of two aromatic rings joined by a three-carbon unit and condensed by an oxygen. The anthocyanin molecule consists of two or three portions, an aglycone (anthocyanidin), a group of sugars, and often a group of organic acids [96].

There are several evidences that relate the role of this flavonoid in cognition and memory, being characterized with a neuroprotective factor in the dementias among them AD. Alim et al. [44] observed in genetically modified mice (A*β*_1–42_ mouse model of AD) the ability of anthocyanin particles and (ethylene glycol) gold nanoparticles (PEG-AuNPs) (polyphenolic flavonoid anthocyanins for conjugation to PEG-AuNPs) to improve memory loss and synaptic deficit and neurodegeneration. The behavioral assessment showed that the latency times of there quired to reach the hidden platform in were shorter, increased the number of platform crossings and time spent in the target quadrant during the probe test in mice treated with anthocyanins and anthocyanin-loaded PEG-AuNPs. Furthermore, anthocyanins and anthocyanin-loaded PEG-AuNPs increased the spontaneous alteration behavior. Anthocyanin-loaded PEG-AuNPs reduced the levels of A*β* (*β*-amyloid protein), BACE-1 (a beta-secretase, a key enzyme in the formation of the *β*-amyloid protein), and APP (protein precursor antiamyloid). This demonstrates a potential action of this flavonoid on the production of beta-amyloid protein. The administration of free anthocyanins and anthocyanin-loaded PEG-AuNPs mitigated the effect of A*β*_1–42_ and increased the expression levels of synaptophysin, PSD95, and SNAP23; these molecules are related to the synapse process between the neurons. Anthocyanins and anthocyanin-loaded PEG-AuNPs increased the phosphorylation of GluR1 at Ser845 and increased the expression level of p-CREB (Ser133), which can improve the memory process. The administration of free anthocyanins and anthocyanin-loaded PEG-AuNPs increased phosphorylation and elevated the levels of p-PI3K and p-Aktat Ser473, increased the level of p-GSK3*β* at Ser9, and reduced the level of p-tau at Ser413 and Ser404, which consequently may reduce the level of formation of fibrillar components. The results showed a reduction of the ratio of Bax/Bcl2 and Cyt c, but anthocyanin-loaded PEG-AuNPs were more effective than free anthocyanin. A reduction in the caspase-9, cleaved caspase-3, and PARP-1 levels in the hippocampus was also observed, and these results demonstrate the neuroprotective action of this flavonoid. Finally, an increase in the number of surviving neurons and a reduction in the A*β*_1–42_-induced-degenerated neuronal cells in the hippocampus and cortex were seen [44].

In the work of Kim et al. [53], the therapeutic efficacy of anthocyanins alone and anthocyanin-loaded PEG-AuNPs in the A*β*_1–42_-induced AD mouse model was also investigated. It was observed that the anthocyanin-loaded PEG-AuNPs can cross the blood-brain barrier and accumulate in the A*β*-injected mice. Furthermore, the anthocyanin-loaded PEG-AuNPs reduced *β*-amyloid and BACE-1 expressions and also prevented tau hyperphosphorylation GSK-3*β*/CDK5 pathway. Anthocyanin-loaded PEG-AuNPs also reduced A*β*_1–42_-induced microglia and astrocyte cell activation [53].

In another study, the anthocyanins inhibited activated astrocytes and various inflammatory markers including p-NF-*κ*B, inducible nitric oxide synthase (iNOS), and tumor necrosis factor-alpha (TNF-*α*) in the hippocampus and cortex regions of D-gal-treated rats [52].

Anthocyanins are able to inhibit the cascade of myeloid beta-protein production and to decrease synaptogenesis and neuronal death. They also induce microglial activation in areas important to the process of memory as hippocampus and cortex.

#### 3.6.4. Naringin

Naringin (4′,5,7-trihydroxyflavanone 7-rhamnoglucoside) belongs to a family of C_6_-C_3_-C_6_ polyphenol compounds and exists in grapefruit and other citrus fruits. This flavonoid has been shown to possess numerous biological benefits such as antioxidant and anti-inflammatory [97]. Preclinical models of atherosclerosis, cardiovascular disorders, diabetes mellitus, neurodegenerative disorders, osteoporosis, and rheumatological disorders were established in a few studies of naringin *in vitro* and *in vivo* [98]. Recently, studies demonstrated a neuroprotective effect of naringin by modulation of endogenous biomarkers and downregulation of free radical and cytokines, including tumor necrosis factor-*α* (TNF-*α*) in streptozotocin-induced painful diabetic neuropathy [99]. Mani et al. [37] investigated the effect of naringin against deltamethrin-induced neurotoxicity in male Wistar rats. The result showed that treatment leads to a significant revival of the oxidative status, which confirms the protective effect of naringin.

Behavioral analysis of the effect of naringin on memory deficit in a pharmacological model (donepezil and scopolamine) in animals has demonstrated a significant difference in the locomotor activity and confirmed that naringin has no confounding influence on locomotion on improving the potential for episodic memory, in the familiarization trial no preference or discrimination toward any of the objects used. The flavonoid reversed the time-induced episodic memory deficit increase in novel object exploration time compared with familiar object and improvement in recognition and discriminative indices. Therefore, naringin reversed the scopolamine-induced short-term episodic memory deficits and improved discrimination and recognition [45].

In a model of animal excitotoxicity by treatment with kainic acid (KA), a potent agonist of excitatory amino acids, especially glutamate, the prevention effect of autophagy and neuroinflammation of naringin was investigated [55]. Excess KA becomes a neurotoxin leading to neuronal death due to excitotoxicity. The naringin treatment significantly decreased the frequency of chronic spontaneous seizures in KA-treated mice compared with KA alone suggesting that naringin might have beneficial properties as an antiepileptic agent. Additionally, treatment with naringin attenuated the loss of hippocampal neurons in the KA-treated CA1 region, suggesting that naringin might have a property of reducing autophagic stress, which could be involved in neuronal cell death. In addition, the naringin treatment attenuated an increase in TNF-*α* within Iba1-positive microglia in the KA-treated hippocampus characteristic of diseases such as Parkinson's and Alzheimer's [55].

The naringenin (5,7-dihydroxy-2-(4-hydroxyphenyl)chroman-4-one) is a flavonoid of citrus fruits, predominantly found in grapefruit. The antinociceptive, anti-inflammatory, and antioxidant effects of the naringenin have already been demonstrated [100]. Lou et al. [58] investigated the effect of naringenin on PD through the pharmacological parkinsonism model (6-OHDA). Naringenin treatment resulted in an increase in nuclear factor E2-related factor 2 (Nrf2) protein (regulatory factor of the expression of antioxidant protein genes) levels and subsequent activation of antioxidant response element (ARE) pathway genes. Additionally, the pretreatment with naringenin protected mice against 6-OHDA-associated ROS damage in striatum. The 6-OHDA-induced loss of TH-positive neurons in the striatum and SNC were remarkably attenuated by naringenin treatment. Therefore, there was a profound reduction in striatal DA and its metabolites after 6-OHDA lesioning that was attenuated by naringenin treatment, which produced a significant elevation in striatal DA and the metabolites DOPAC (dihydroxyphenylacetic acid) and HVA (homovanillic acid). Furthermore, apomorphine-induced asymmetrical rotations contralateral to the 6-OHDA injection site were significantly reduced by naringenin treatment as compared to mice lesioned with 6-OHDA [58].

Narigin seems to act especially through the inhibition of oxidative cellular stress, which reflects in reduction of neuronal loss (autophagic stress). It is known that oxidative stress is a strong mechanism of neuroinflammation and has an important consequence for neurodegenerative diseases.

#### 3.6.5. Baicalein

Baicalein (5,6,7-trihydroxyflavone) is a flavonoid originally isolated from the roots of *Scutellaria baicalensis* and *Scutellaria lateriflora* [101]. It has neuroprotective properties against PD and antioxidant and anti-inflammatory properties [102]. Hu et al. [63] noted that baicalein treatment attenuated the motor deficits, in addition to increase in striatal neurotransmitters: DA (dopamine), DOPAC (3,4-dihydroxyphenylacetic acid), and HVA (homovanillic acid). Analysis of fluorescence intensity by microscopy and the amount of *α*-syn in enteric nervous system was also lower and increased of the in the number of TH-positive neurons. There was a decrease of *α*-synoligomers, not monomers, in ileum, thoracic spinal cord, and midbrain; baicalein had no effect on *α*-syn mRNA expression. Therefore, it can be inferred that this flavonoid can prevent the progression of *α*-syn accumulation in PD, by inhibiting the formation of *α*-syn oligomers [63].

When investigating the therapeutic effects of baicalein on rotenone-induced PD rats and exploring whether the neuroprotective potential practices by baicalein were through intervening in mitochondrial function and mitobiogenesis, it was found that baicalein partially ameliorated the motor dysfunction and increased the number of TH+ cells in the substance nigra (SN) in rotenone-induced PD rats [64]. This flavonoid also protected neurons in the SN against rotenone-induced apoptosis. The baicalein ameliorated the dysfunction of mitochondrial complex I in the ventral midbrain that was damaged by rotenone [64]. In addition, the administration of baicalein increased the protein levels of PGC-1*α* (a regulator of mitochondrial mitobiogenesis), NRF-1 (transcription factor that regulates the expression of antioxidant proteins), and TFAM (mitochondrial transcription factor) in the ventral midbrain, which may improve the brain's response to oxidative stress and consequent neuronal loss observed in Parkinson's disease [64].

Lee et al. [56], through a model of pharmacological parkinsonism in animals (MPTP administration), observed that the, in low doses, baicalein improves motor ability and prevented the loss of dopaminergic neurons caused by MPTP. In addition, microglial activation and astrocyte activation were reduced in the animal with pretreated baicalein PD. This study reveals the importance of astrocyte activation for the occurrence of the neurodegenerative process. It has also been reported that baicalein reduces MPP (a toxic molecule that interferes with oxidative phosphorylation in mitochondria) that is capable of inducing the activation of NF-*κ*B, ERK (protein kinase intracellular signaling), and JNK (c-jun N-terminal kinase) in the astrocyte leading to a mechanism of neuroinflammation considered as a potent inducer of PD [56].

These studies taken together reveal the neuroprotective action of baicalein especially on mitochondrial activity and activation of glial cells. Both processes are recognized with potential mechanisms capable of increasing the risk of neurodegenerative diseases. This flavonoid proved to be effective as a therapeutic strategy especially against PD disease.

#### 3.6.6. Hesperidin

Hesperidin, (2*S*)-5-hydroxy-2-(3-hydroxy-4-methoxyphenyl)-4-oxo-3,4-dihydro-2*H*-chromen-7-yl 6-*O*-(6-deoxy-*α*-*L*-mannopyranosyl)-*β*-*D*-glucopyranoside, is the major flavanone glycoside present in citrus fruits. This compound has an important neuroprotective property and radical scavenging properties related to diverse neuronal insults, such as ischemia [103], stroke [104], and oxidative-induced damage [105], as well as pathology related to AD [106] and Huntington's disease [107].

Nones et al. [108] showed that hesperidin promotes neuronal differentiation and survival and also enhances the neuroprotective capacity of astrocytes, by inducing them to secrete soluble factors involved in neuronal survival *in vitro*.

Li et al. [40] investigated the potential therapeutic effect of hesperidin on behavioral dysfunction, A*β* deposition, and neuroinflammation in the transgenic APP/PS1–21 mouse model. In this research, the treatment with hesperidin caused a decrease in the nesting ability and social interaction. Furthermore, the improvement of amyloid beta accumulation and APP expression, with reduction of microglial activation, suggests that hesperidin might be a potential candidate for the treatment of AD or even of other neurodegenerative diseases.

An investigation into the anti-inflammatory potential, antioxidants, and protective effects of hesperidin was performed by Javed et al. [41] using the mouse model of sporadic dementia of Alzheimer's type (SDAT). In this model, researchers have shown that hesperidin can be used for the treatment of cognitive disorders because of its neuronal cell death modulation by inhibiting the overexpression of inflammatory markers like nuclear factor *κ*B, coupled with the inducible nitric oxide synthase, cyclooxygenase-2, and glial fibrillary acidic protein-positive astrocytes.

Antunes et al. [57] evaluate the role of the flavonoid hesperidin in an animal model of PD induced by 6-OHDA and demonstrated that hesperidin (50 mg/kg) treatment was effective in preventing memory impairment and depression-like behavior with reduction in glutathione peroxidase and catalase activity, total reactive antioxidant potential, and the dopamine and its metabolite levels in the striatum of aged mice.

Matias et al. [109] described hesperidin as a new drug able to improve memory in healthy adult mice by two main mechanisms: by inducing synapse formation and function between hippocampal and cortical neurons. In addition, other mechanisms enhance the synaptogenic ability of cortical astrocytes by means of increased secretion of transforming growth factor beta-1 (TGF-*β*1) by these cells.

Hesperidin appears to present a neuroprotective role through its action on glial cells, microglia, and astrocytes, promoting reduction of neuroinflammation and oxidative stress. In addition, it may induce synapse formation in brain regions involved with memory and decision-making.

#### 3.6.7. Rutin

Rutin (2-(3,4-dihydroxyphenyl)-5,7-dihydroxy-4-oxo-4*H*-chromen-3-yl 6-*O*-(6-deoxy-*α*-*L*-mannopyranosyl)-*β*-*D*-glucopyranoside) is a glycone of quercetin with a flavonol structure. This substance modifies the cognitive and various behavioral symptoms of neurodegenerative diseases due to the ability of rutin and/or its metabolites to cross the blood-brain barrier; this way causes effects on the various cellular functions under pathological conditions [110, 111].

Some reports have demonstrated that rutin scavenges superoxide radicals, increases antioxidant enzymatic activity *in vitro*, reduces lipid peroxidation and cytokine production, and prevents cognitive deficits including CNS injuries in rat models [112, 113].

In recent studies, Rodrigues et al. [114] investigate the effect of treatment with rutin after induction of focal cortical ischemia and results that shown that rutin is a putative candidate to treat stroke. Few studies have evaluated the treatment with rutin in models of global and focal brain ischemia, showing positive effects [115].

According to Hhan et al. [116], similar to other flavonoids, the main expected mechanisms of action of rutin are its anti-inflammatory and antioxidative potentials. Thus, anti-inflammatory action of rutin was demonstrated with reduction of inducible nitric oxide synthase expression in a model of PD.

Moghbelinejada et al. [39] investigated the possible effects of rutin on MAPK and BDNF gene expression and memory retrieval in *β*-amyloid-injected rats. Their results demonstrated improved memory impairment caused by injection of A*β* in rats through activation of MAPK and BDNF, as also reduced oxidative stress in the hippocampus of rats by reducing MDA level and increasing thiol content in the hippocampus. Further studies are necessary to clarify the effects and molecular mechanisms of this flavonoid.

Rutin has antioxidant and anti-inflammatory roles; its mechanism of action is not yet completely elucidated but may be related to the MAPK pathway and reduction of nitric oxide synthase activation.

## 4. Conclusion and Perspective

This systematic review suggests that the flavonoids reported have a potential for the treatment of neurodegenerative diseases such as PD and AD and are considered drug candidates in the future clinical research. The studies listed in this review revealed that the main targets of action for Alzheimer's disease therapy were reduction of reactive oxygen species and amyloid beta-protein production. In Parkinson's disease, reduction of the cellular oxidative potential and mechanisms of neuronal death are often involved in the neuroprotective potential of flavonoids.

It was observed that flavonoids had been studied using various *in vivo* animal models, including the evaluation of its mechanism of action and effects on the molecular level. However, it is essential to improve the rigor of study design and data in view of the fact that most of the included studies presented low to moderate methodological quality, which limits the interpretation of the results and the continuity of the studies.

## Figures and Tables

**Table 1:** Characteristics of studies inserted in the review.

Authors, year, country	Substance(s)	Disease	Animals, (strain/sex) *n* (per group)	Doses, route, administration, period	Preclinical models	Evaluated parameters	
						Behavior	Biochemical/molecular
Wei et al., 2013 [36], China	(2S)-5, 2′, 5′-trihydroxy-7-methoxyflavanone (TMF)	Alzheimer's	Mice (Kunming/NR) (*n* = 8)	4 or 8 mg/kg, i.p., 1 week	D-galactose	(i) Spatial learning and memory (Morris water maze test)	(i) Colorimetric assay: GSH and GSSG (ii) ELISA: AP-1 and BDNF (iii) Western blot: CREB and p-CREB
Mani et al., 2013 [37], India	Naringin	Alzheimer's	Rats (W/M), *n* = 6	100 mg/kg, p.o., 21 days	Deltamethrin	(i) NR	(i) Agarose gel electrophoresis: DNA fragmentation (ii) Biochemical: LDH, CK, AChE, SOD, CAT, GPx, GR, GSH, vitamin C, vitamin E, and lipid peroxidation level (iii) Histological analysis (iv) Native gel electrophoresis: SOD and CAT
Nakajima et al., 2013 [38], Japan	Nobiletin	Alzheimer's	Rats (SAMP8/M) *n* = 47	10 or 50 mg/kg, i.p., unclear	Senescence-accelerated prone mouse (SAMP8) model	(i) Nonspatial memory (novel object test) (ii) Contextual and auditory fear memory (fear conditioning test) (iii) Emotional reactivity, anxiety (elevated plus maze test)	(i) Biochemical: MDA, protein carbonyl level, SOD, and GPx (ii) HPLC: GSH and GSSG (iii) Real-time RT-PCR: GPx1 and GPx4 (iv) Western blot: A*β*_1–42_, tau, p-tau, GPx1, and GPx4
Moghbelinejad et al., 2014 [39], Iran	Rutin	Alzheimer's	Rats (W/M) *n* = 30	100 mg/kg, i.p., 3 weeks	Amyloid beta (A*β*_1–42_)	(i) Memory retrieval (passive avoidance apparatus test)	(i) Biochemical: MDA and total SH groups (ii) Real-time RT-PCR: BDNF, ERK1, ERK2, and CREB1
Li et al., 2014 [40], Germany	Hesperidin	Alzheimer's	Mice (APP/PS1–21/M), *n* = 12	100 mg/kg, p.o., NR	Transgenic APP/PS1–21 mouse model	(ii) Nesting behavior (nest building assay) (iii) Social behavior, degree of interaction (social interaction assay)	(i) Double immunostaining (ii) Immunohistochemistry: A*β*, GFAP, TGF-*β*1, and Alzheimer precursor protein A4
Javed et al., 2014 [41], India	Hesperidin	Alzheimer's	Mice (S/M), *n* = 10 or *n* = 12	100 and 200 mg/kg, i.p, 15 days	Streptozotocin	(i) Spatial learning and memory (Morris water maze test)	(i) Biochemical: TBARS, GSH, AChE, ganglioside, and phospholipids (ii) Immunohistochemistry: GFAP, NF-*κ*B, iNOS, and COX-2
Walker et al., 2015 [42], USA	Epigallocatechin gallate	Alzheimer's	Mice (TgCRND8 (Tg) and wild type (nTg)/M and F), *n* = 10, *n* = 11, or *n* = 12	50 mg/kg, p.o., 4 months	TgCRND8 amyloid precursor protein transgenic mice	(i) Acquisition experience (nest building) (ii) Locomotor activity and exploratory (open field test) (iii) Novelty-seeking and anxiety-like behaviors (light-dark box) (iii) Learning (Barnes maze)	(i) ELISA: A*β*_1–42_
Kou et al., 2016 [43], China	Dihydromyricetin	Alzheimer's	Rats (SD/M), *n* = 10	100 and 200 mg/kg, p.o., 6 weeks	D-galactose	(i) Spatial learning and memory (Morris water maze test)	(i) Histological analysis (ii) Real-time PCR: miR-34a (iii) SA-*β*-gal staining (iv) Western blot: caspase-3, Bcl-2, SIRT1, p53, p21, Atg7, LC3-II/LC3-I, GFAP, and mTOR
Ali et al., 2016 [44], Republic of Korea	Anthocyanins and anthocyanin-loadedPEG-AuNPs	Alzheimer's	Mice (C57BL/M), *n* = 15	12 *μ*g/g/day, p.o., 14 days	Amyloid beta (A*β*_1–42_)	(i) Spatial learning and memory (Morris water maze and Y-maze tests)	(i) Immunohistochemical Nissl and FJB staining (ii) Immunofluorescence (iii) Western blot: A*β*, BACE-1, SNAP23, synaptophysin, p-AMPARs, p-PI3K, p-Akt, p-GSK3*β*, p-tau, PSD95, caspase-3, Cyt c, Bax, Bcl2, and poly (ADP-ribose) polymerase-(PARP-1)
Ramalingayya et al., 2016 [45], India	Naringin and Rutin	Alzheimer's	Rats (W/F), *n* = 12	50 and 100 mg/kg, p.o., 15 days	Donepezil and scopolamine	(i) Locomotor activity and time spent in the center zone (open field test) (ii) Nonspatial memory (object recognition task)	(i) Hematological
Chen et al., 2016 [46], China	Quercetin	Alzheimer's	Mice (C57BL/6J/M), NR	30 mg/kg, p.o., NR	Cognitive disorders per high-fat diet (HFD)	(i) Spatial learning and memory (Morris water maze test)	(i) Immunohistochemisty: p-PERK, p-IRE1*α*, NLRP3, and p-tau (ii) Light microscopy: CA1 (iii) Western blot: AMPK, p-AMPK, IRE1*α*, p-IRE1*α*, eIF-2*α*, p-IF-2*α*, TXNIP, NLRP3, GSK-3*β*, p-GSK-3*β*ser9, tau, and p-tau
Song et al., 2017 [47], China	Silibinin	Alzheimer's	Rats (SD/M), NR	25, 50, and 100 mg/kg, p.o., 28 days	Amyloid beta (A*β*_25–35_)	(i) Anxiety and locomotor activity (Elevated plus maze test) (ii) Spatial learning and memory ability (Morris water maze test) (iii) Learning and memory (novel object-recognition test) (iv) Memory (memory flexibility test)	(i) Biochemical: MDA and GSH (ii) ELISA: IL-1*β*, IL-4 (iii) Flow cytometric analysis (iv) Transmission electron microscopy (v) Western blot: NF-*κ*B, COX-2, iNOS, p53, and p-p53
Moreno et al., 2017 [48], Spain	Quercetin and quercetin-loadednanoparticles	Alzheimer's	Rats (SAMP8/M), *n* = 8	Quercetin (25 mg/kg, p.o., 2 months) Quercetin-loaded nanoparticles (25 mg/kg every 48 hours, p.o., 2 months)	Senescence-accelerated prone mouse (SAMP8) model	(i) Locomotor activity (open field test) (ii) Motor coordination and balance (rotarod test) (ii) Exploratory motivation (marble burying test) (iii) Spatial memory and both the working and reference memory functions (Morris water maze test)	(i) Western blot: GFAP and CD11b
Godoy et al., 2016 [49], Chile	Quercetin	Alzheimer's	Rats (B6.129S7-Sod2tm1Leb/J/NR), NR	50 mg/kg, two times a week, p.o., 4 weeks	Amyloid beta (A*β*_25–35_)	(i) NR	(i) Electrophysiology
Palle and Neerati, 2016 [50], India	Quercetin and quercetin nanoparticles	Alzheimer's	Rats (W/M), *n* = 6	30 mg/kg, i.p., 8 days	Scopolamine	(i) Conditioning, avoidance responses (conditioned avoidance test) (ii) Learning, memory (rectangular maze test)	(i) Biochemical: MDA, GPx, AChE, and CAT (ii) Histological analysis
Ahmad et al., 2016 [51], Republic of Korea	Fisetin	Alzheimer's	Mice (C57BL/6N/M), *n* = 12	20 mg/kg, i.p., 2 weeks	Amyloid beta A*β*_1–42_	(i) Spatial memory and both the working and reference memory functions (Morris water maze test)	(i) FJB staining (ii) Immunofluorescence: A*β* (B4), synaptophysin, PSD95, p-tau, GFAP, and Iba-1 (iii) Immunohistochemistry: caspase-3 (iv) Western blot: caspase-9, SYN, p-AMPAR1, p-CREB, p-CAMKII, p-PI3K, and p-Akt
Rehman et al., 2017 [52], Republic of Korea	Anthocyanins	Alzheimer's	Rats (SD/M), *n* = 13	100 mg/kg, i.p., 7 weeks	D-galactose	(i) Spatial learning and memory (Morris water maze and Y-maze tests)	(i) Biochemical: ROS, MDA (ii) Immunofluorescence: A*β*, 8-OxoG, p-JNK, GFAP, and Iba-1 (iii) Western blot: A*β*, BACE-1, RAGE, 8-OxoG, TNF-*α*, iNOS, p-JNK, Bax, Bcl2, PARP-1, syntaxin, synaptophysin, SNAP-23, p-CREB, GFAP, and Iba-1
Kim et al., 2017 [53], Republic of Korea	Anthocyanins alone and anthocyanin-loaded poly (ethylene glycol) gold nanoparticles (PEG-AuNPs)	Alzheimer's	Mice (C57BL/6N/M), *n* = 8	10 mg/kg, i.v., 14 days	Amyloid *β* (A*β*_1–42_)	(i) NR	(i) ICP-AES (ii) Immunofluorescence: GFAP, Iba-1, and RAGE (iii) Nissl staining (iv) TEM (v) Western blot: A*β*, BACE-1, GSK3*β*, CDK5, tau, NF-*κ*B, iNOS, p-JNK, Bcl2, Bax, Cyt c, FJB, COX-2, NOS3, IL-1*β*, and TNF-*α*
Sharma et al., 2016 [54], India	Quercetin	Alzheimer's and Parkinson's	Rats (W/M), *n* = 6	10 mg/kg, p.o., 12 weeks	Aluminum	(i) NR	(i) Biochemical: ROS, MnSOD (ii) DNA isolation for DNA fragmentation (iii) Electron microscopy analysis (iv) Histological analysis (v) Immunohistochemistry: MnSOD, c-c, and caspase-3 (vi) RT-PCR: MnSOD; (vi) Western blot: MnSOD, Cyt c, Bax, Bcl-2, p53, and caspase-3
Jeong et al., 2015 [55], Republic of Korea	Naringin	Epilepsy, Parkinson's, and Alzheimer's	Mice (C57BL/6/M) NR	80 mg/kg, i.p., 7 days	Kainic acid	(i) NR	(i) Immunohistochemical: NeuN and Iba-1 (ii) Light microscopy: CA1 (iii) Western blot: LC3B and TNF*α*
Lee et al., 2014 [56], Republic of Korea	Baicalein	Parkinson's	Mice (C57B/6/M), *n* = 8–14	1 and 10 mg/kg (i.p.), 7 days	MPTP	(i) Motor coordination and balance (rotarod test)	(i) DAB immunostaining: TH (ii) Double label immunostaining: TH, GFAP, and Iba-1 (iii) Histological analysis (iv) Western blot: GFAP
Antunes et al., 2014 [57], Brazil	Hesperidin	Parkinson's	Mice (C57 BL/6/F), *n* = 10	50 mg/kg, p.o., 28 days	6-OHDA	(i) Depression (Tail-suspension test) (ii) Spatial learning and memory (Morris water maze) (iii) Locomotor activity and time spent in the center zone (open field test)	(i) Biochemical: GSH, ROS, TRAP, SOD, CAT, GR, GPx, GST, DA, DOPAC, and HVA
Lou et al., 2014 [58], China	Naringenin	Parkinson's	Mice (C57BL/6/F), *n* = 10	70 mg/kg, p.o., 4 days	6-OHDA	(i) Rotational behavior—numbers of rotations (apomorphine-induced circling behavior)	(i) HPLC-MS: DA, DOPAC, and HVA (ii) Immunohistochemistry: TH (iii) Western blot: Nrf2, HO-1, GCLM, GCLC, Lamin A, cleaved caspase-3, p-JNK, JNK, p-p38, and p38
Wang et al., 2015 [59], China	Tanshinone I	Parkinson's	Mice (C57BL/6/M), NR	5, 10 mg/kg, p.o., 7 days	MPTP	(i) Motor coordination and balance (rotarod test)	(i) Biochemical: ALT, AST, and ALP (ii) ELISA: TNF-*α* and IL-10 (iii) HPLC: DA, DOPAC, HVA, and MPP+ (iv) Immunohistochemistry: TH and Iba-1
Chen et al., 2015 [60], China	Silibilin	Parkinson's	Rats (M and F), NR	25 and 50 mg/kg, p.o. in second day	Increased neonatal iron intake	(i) Locomotor activity and time spent in the center zone (open field test) (ii) Motor coordination and balance (rotarod test)	(i) Biochemical: MDA and GSH (ii) HPLC-ECD: DA and 5-HT
Mu et al., 2016 [61], China	Quercetin	Parkinson's	Rats (SD/M), NR	100, 200, and 400 mg/kg, i.g., NR	6-OHDA	(i) Rotational behavior—numbers of rotations (apomorphine-induced circling behavior)	(i) HPLC-ECD: DA, DOPAC, HVA, 5-HT, and 5-HIAA
Lee et al., 2015 [62], Korea	Silibinin	Parkinson's	Mice (C57B/6/M), *n* = 10 or 12	1 or 10 mg/kg, i.p., 5 days	MPTP	(i) Motor coordination and balance (rotarod test)	(i) DAB immunostaining: TH (ii) Double label immunostaining: GFAP and Iba-1
Hu et al., 2016 [63], China	Baicalein	Parkinson's	Mice (C57BL/6/M), NR	100 mg/kg, i.p., 7 weeks to 12 weeks	Rotenone	(i) Motor coordination and balance (rotarod test) (ii) Motor dysfunctions (grid test)	(i) HPLC: DA, DOPAC, and HVA (ii) Immunofluorescence: *α*-syn, TH, and ChAT (iii) Real-time PCR: *α*-syn (iv) TEM (v) Western blot: *α*-synuclein and GAPDH
Zhang et al., 2017 [64], China	Baicalein	Parkinson's	Rats (SD/M), *n* = 15, or 10	100, 200, and 400 mg/kg, p.o., 28 days	Rotenone	(i) Locomotor activity (open field test) (ii) Motor coordination and balance (rotarod test) (iii) The inclined plane assessment	(i) Immunohistochemistry: TH (ii) TEM (iii) TUNEL staining (iv) Western blot: caspase-3, PGC-1*α*, NRF-1, and TFAM
Goes et al., 2017 [65], Brazil	Chrysin	Parkinson's	Mice (C57B/6J/M), *n* = 6	10 mg/kg, p.o., 28 days	6-OHDA	(i) Motor coordination and balance (rotarod test) (ii) Rotational behavior—numbers of rotations (apomorphine-induced circling behavior)	(i) ELISA: IFN-*γ*, TNF-*α*, IL-6, IL-10, NF-*κ*B, S100B, BDNF, GDNF, and NGF (ii) HPLC: DA, DOPAC, and HVA (iii) TRAP and TAR (iv) Immunohistochemistry: TH^+^ neurons
Ay et al., 2017 [66], USA	Quercetin and quercetin-containingformulation (QB3C)	Parkinson's	Mice (MitoPark and C57BL/6/M/F), *n* = 8 or *n* = 9	Quercetin (25 mg/kg, p.o., 6 weeks) QB3C comprising quercetin (175 mg/kg, p.o., 8 weeks)	MitoPark transgenic mouse models	(i) Locomotor activity (open field test) (ii) Motor coordination and balance (rotarod test)	(i) HPLC: DA, DOPAC, and HVA (ii) DAB immunostaining: TH

Animals: SD: Sprague-Dawley; W: Wistar; S: Swiss; SAMP8: senescence-accelerated prone mouse 8; NR: not reported. Parameters assessed: DPPH: 2,2-diphenyl-1-picrylhydrazyl radical; MDA: malonaldehyde; TBARS: thiobarbituric acid reactive substances; AAPH: 2,2′-azobis(2-methylpropionamidine) dihydrochloride; FeSO4: ferrous sulphate; 6-OHDA: 6-hydroxydopamine; MPTP: 1-methyl-4-phenyl-1,2,3,4-tetrahydropyridine; FJB: Fluoro-Jade B; GSH: reduced glutathione; GSSG: oxidized glutathione; AP-1: activator protein-1; BDNF: brain-derived neurotrophic factor; CREB: cAMP response element-binding protein; p-CREB: phosphorylated; SOD: superoxide dismutase; CAT: catalase; GPx: glutathione peroxidase; GR: glutathione reductase; GSH: reduced glutathione; LDH: lactate dehydrogenase; CK: creatine kinase; AChE: acetylcholinesterase; MDA: malondialdehyde; p-tau: phosphorylated tau; TGF-*β*1: transforming growth factor beta 1; SYN: synaptophysin; p-AMPAR1: phospho-*α*-amino-3-hydroxy-5-methyl-4-isoxazolepropionic acid receptors; p-CRE: phosphorylated cAMP response element binding protein; p-CAMKII: phosphorylated calcium/calmodulin-dependent protein kinase II; p-PI3K: phosphorylated phosphatidylinositol-4,5-bisphosphate 3-kinase; p-Akt: phosphorylated protein kinase B; GFAP: antiglial fibrillary acidic protein; Iba-1: anti-ionized calcium-binding adapter molecule 1; 8-OxoG: 8-oxoguanine; p-JNK: C-jun N-terminal kinase; ICP-AES: inductively coupled plasma-atomic emission spectrometer; TEM: transmission electron microscopy; A*β*: brain expression levels of amyloid beta; BACE-1: beta-site APP cleaving enzyme 1; GSK3*β*: glycogen synthase kinase-3*β*; CDK5: cyclin-dependent kinase 5; GFAP: glial fibrillary acidic protein; NF-*κ*B: nuclear factor kappa B; iNOS: inducible nitric oxide synthase; COX-2; NOS3; IL-1*β*; TNF-*α*; p-JNK: phospho-JNK; Bcl2; Bax; Cyt c: cytochrome c; FJB. RAGE receptor for advanced glycation end products; MnSOD: mitochondrial superoxide dismutase; NeuN: neuronal nuclei; LC3B: microtubule-associated protein light chain 3 isoform B; MS: mass spectrometry; DA: dopamine; DOPAC: dihydroxyphenylacetic acid; HVA: homovanillic acid; HO-1: hemeoxygenase; Nrf2: nuclear factor E2-related factor 2; GCLC: glutathione cysteine ligase regulatory subunit; GCLM: glutathione cysteine ligase modulatory subunit; JNK: c-Jun N-terminal kinase; MPP+: 1-methyl-4-phenylpyridinium; ALT: alanine aminotransferase; AST: aspartate aminotransferase; ALP: alkaline phosphatase; HPLC: high-performance liquid chromatography; ECD: equipped with electro chemical detector; TRAP: total reactive antioxidant potential; TAR: total antioxidant reactivity; S100B: calcium-binding protein B; GDNF: glial cell line-derived neurotrophic factor; NGF: nerve growth factor; DAB: diaminobenzidine; *SA*-*β*-*Gal*: senescence-associated *β*-*galactosidase.*
